# Increased risk of alcohol use disorder following bariatric surgery: literature review

**DOI:** 10.1192/j.eurpsy.2025.429

**Published:** 2025-08-26

**Authors:** P. Alves Peixoto, A. F. Teixeira

**Affiliations:** 1 Departamento de Psiquiatria e Saúde Mental, Unidade Local de Saúde do Tâmega e Sousa, Penafiel; 2 Centro de Respostas Integradas de Braga – Equipa de Tratamento de Guimarães, Instituto para os Comportamentos Aditivos e as Dependências, Guimarães, Portugal

## Abstract

**Introduction:**

In the last 50 years, obesity has become a leading cause of morbidity and decreased life expectancy, being associated with the development of cardiovascular disease, decreased mental health and overall decreased quality of life. The development of bariatric surgery as a treatment strategy in those in which attempts at conservative treatment yielded no satisfactory results has revolutionized treatment of refractory obesity. While short term gains in cardiovascular health are undeniable, long-term impact remains uncertain. More recently, there is emerging evidence of bariatric surgery being associated with *de novo* alcohol use disorder.

**Objectives:**

We aimed to study the possible correlation between bariatric surgery and alcohol use disorder, specifically the individual risk factors that may promote such outcome. Moreover, we aimed to evaluate which surgical procedures are more eliciting of alcohol use disorder.

**Methods:**

We performed a literature review using the database MEDLINE and obtained 241 results using the query terms “*bariatric surgery*” and “*alcohol*”. Of these, we read all the summaries and subsequently selected 51 different scientific articles which we afterwards read in full. We then performed a literature review aiming to understand the maladaptive alcohol consumption patterns following bariatric surgery

**Results:**

Most studies report maladaptive alcohol use following bariatric surgery. Outstandingly, this consumption pattern develops only at later follow up stages, usually over 3 years after surgery. Furthermore, not all types of bariatric surgery appear to pose the same iatrogenic risk. Gastric bypass poses the highest risk of new onset alcohol misuse, in comparison to sleeve gastrectomy or gastric band surgery. It is not yet fully understood the mechanism behind this pattern of alcohol misuse following surgery, but common reasons have been identified, including different social interaction patterns, addiction transfer, ambivalence towards bodily changes, increased mental vulnerability and different body response to the effects of alcohol.

**Conclusions:**

The screening of alcohol misuse before and after bariatric surgery is of paramount importance to decrease the surgical iatrogenic risk and improve outcomes. Further research should be developed in understanding what are the risk factors and mechanistic pathways for alcohol misuse following bariatric surgery. Also, it should be investigated whether the treatment of alcohol misuse disorder differs between patients submitted to bariatric surgery or not.

**Disclosure of Interest:**

None Declared

